# Genetic Adaptation of Porcine Circovirus Type 1 to Cultured Porcine Kidney Cells Revealed by Single-Molecule Long-Read Sequencing Technology

**DOI:** 10.1128/genomeA.01539-16

**Published:** 2017-02-02

**Authors:** Dóra Tombácz, Norbert Moldován, Zsolt Balázs, Zsolt Csabai, Michael Snyder, Zsolt Boldogkői

**Affiliations:** a Department of Medical Biology, Faculty of Medicine, University of Szeged, Szeged, Hungary; b Department of Genetics, School of Medicine, Stanford University, Stanford, California, USA

## Abstract

Porcine circovirus type 1 (PCV1) is a nonpathogenic circovirus, and a contaminant of the porcine kidney (PK-15) cell line. We present the complete and annotated genome sequence of strain Szeged of PCV1, determined by Pacific Biosciences RSII long-read sequencing platform.

## GENOME ANNOUNCEMENT

Porcine circovirus type 1 (PCV1) is a member of the *Circoviridae* family. The 16 to 21 nm virion was described by Tischer and coworkers in 1974 (1) as a contaminant particle of the epithelial cell line from porcine kidney (PK-15; ATCC CCL-33). The genome of PCV1 is composed of a 1,759 nucleotide (nt) long, single-stranded, circular genome (2). Although PCV1 is nonpathogenic in adult swine, it was shown to cause lesions in experimentally infected pig fetuses (3). The genetic variation of PCV1 isolated from swine is relatively low, suggesting a high adaptation of PCV1 to its host (4). The porcine circovirus type 2 (PCV2) is associated with post-weaning multisystemic wasting syndrome and other porcine circovirus-associated diseases (PCVAD) (5) in swine. The single origin of DNA replication (Ori) of PCV1 forms a “stem-loop” structure. The two main open reading frames (ORF1 and ORF2) stand in a divergent orientation relative to each other and are separated by the Ori. The rep gene encodes two replication initiation protein isoforms, Rep and Rep′, the cap gene codes for the capsid protein (Cap) (6). PCV1 was isolated from the porcine kidney cell line PK-15 (purchased from the American Type Culture Collection more than 30 years ago). The purified dsDNA was sequenced by using Pacific Biosciences RSII platform. SMRTbell template libraries were prepared from the DNA using standard protocols, as previously described (7, 8). Sequencing was performed in five single-molecule real-time (SMRT) cells with P5 DNA polymerase and C3 chemistry (P5-C3) yielding a total of 68 reads and a genome coverage of 48.37× on average (ranging from 42× to 57×). The average length of the ROIs was 1,170.661 nt (median 1,168.5). Reads were processed and mapped to the genomic reference sequence of PCV1 (GenBank accession no. NC_001792.2) with the Pacific Biosciences SMRT analysis pipeline (http://www.pacb.com/products-and-services/analytical-software/devnet/) and GMAP (9). The genome of PCV1 strain Szeged is characterized as a single-stranded circular DNA composed of 1,759 bps, with an average G+C content of 48.44% and contains two protein-coding genes. The PCV1 strain Szeged differs in nine point mutations and one insertion mutation from the NCBI reference sequence. Comparison of the DNA sequences available from the NCBI nucleotide database reveals a genetic adaption to the conditions in a cell culture, since the proportion of the nonconservative changes to the silent mutations are relatively high (KN/KS = 1.89). Additionally, heterogeneity was detected in three genomic locations: at position 67 C is substituted with G (ratio: 33%); at position 1,105 C is substituted to T (ratio: 43%); at position 1,503 C is substituted to T (ratio: 32%).

### Accession number(s).

The complete and annotated genome sequence of PCV1 strain Szeged has been deposited in GenBank under accession no. KX816645.
